# Comparison of volar-flexion, ulnar-deviation and functional position cast immobilization in the non-operative treatment of distal radius fracture in elderly patients: a pragmatic randomized controlled trial study protocol

**DOI:** 10.1186/s12891-017-1759-y

**Published:** 2017-09-18

**Authors:** Lauri Raittio, Antti Launonen, Teemu Hevonkorpi, Toni Luokkala, Juha Kukkonen, Aleksi Reito, Bakir Sumrein, Minna Laitinen, Ville M. Mattila

**Affiliations:** 1 University of Tampere, School of Medicine, 33014 Tampere, Finland; 2 Department of Orthopaedics, Unit of Musculoskeletal Surgery, Tampere University Hospital, Teiskontie 35, PL2000, 33521 Tampere, Finland; 3 Central Finland Central Hospital, Keskussairaalantie 19, 40620 Jyväskylä, Finland; 4 Satakunta Central Hospital, Sairaalantie 3, 28500 Pori, Finland

**Keywords:** Colles’ fracture, Rehabilitation, Conservative treatment, Treatment outcome, Prospective studies, Pain, Clinical protocols

## Abstract

**Background:**

Distal radius fractures (DRFs) are the second most common fractures, after hip fractures, seen in clinical practice. The high incidence of low-energy trauma DRFs in elderly patients raises questions about the best treatment method in terms of function, pain, and quality of life. Although the majority of these fractures are treated non-operatively with cast immobilization, valid scientific evidence of the optimal cast immobilization is lacking. In addition, several publications, including Cochrane review have outlined the need for more evidence to determine the most appropriate method of cast immobilization.

**Methods:**

This study is a pragmatic, prospective, randomized, multi-centre trial. The trial is designed to compare two widely used cast positions (volar flexion-ulnar deviation position and functional position) for the non-operative treatment of DRF in patients over 64 years of age. The main hypothesis of the trial is that function position yields corresponding functional outcome, pain relief and quality of life when compared to the volar flexion-ulnar deviation position. The primary outcome measure is Patient Rated Wrist Evaluation (PRWE) score and the secondary outcome measures will be the Disabilities of the Arm, Shoulder and Hand (DASH) score, Visual Analogue Scale (VAS), 15-dimensional (15D) value and rate of surgical interventions. The results of the trial will be analysed after 1 and 2-years.

**Discussion:**

This publication presents a prospective, pragmatic, randomized, national multi-centre trial study protocol. It provides details of patient flow, randomization, follow-up and methods of analysis of the material as well as publication plan.

**Trial registration:**

ClinicalTrials.gov identifier: NCT02894983 22 August 2016.

**Electronic supplementary material:**

The online version of this article (10.1186/s12891-017-1759-y) contains supplementary material, which is available to authorized users.

## Background

Distal radius fractures (DRFs) are prevalent in the general population, and in patients over 60 years of age they are the second most common fractures after hip fractures. [1]. In elderly patients, DRFs are typically caused by a fall from a standing height over an outstretched hand, whereas in younger patients the fracture is mainly caused by high-energy trauma such as motor vehicle collisions [2, 3]. The population-based fracture incidence varies between nations. In Northern Europe, the overall incidence is 200–320/100,000 person years and 550/100,000 person years in patients over 60 years of age [4–7]. The overall fracture incidence of DRFs in the Nordic countries seems to have levelled off in the last two decades [8].

The majority of DRFs are treated with cast immobilization. The theoretical purpose of the cast is to stabilize the fracture, and thus to allow the bone to heal. During cast immobilization, the stabilized fracture maintains alignment. However, a significant proportion of fractures will lose alignment during cast immobilization, >especially in older patients with osteoporotic bone. The alignment of these unstable fractures is often acceptable after closed reduction and at the beginning of cast immobilization, but is lost during the period the cast is worn [9].

Several different immobilization methods for the non-operative treatment of DRFs have been described [10–15]. These methods of immobilization include functional bracing, the immobilization of the wrist in neutral and slightly extended position or in pronation or in supination. The original reduction into volar flexion- was first described by Frederic J. Cotton in 1910 [16]. The slight flexion-ulnar deviation- position was popularized by Charnley in his seminal work [17]. It has been thought that a slight flexion-ulnar deviation position would induce soft tissue around the fracture and the pull produced by radiocarpal ligaments (i.e. ligamentotaxis) would resist the dislocating forces generated over the fracture line [18, 19].

In clinical practice, the flexion-ulnar deviation position can cause the common extensor tendons to tighten and produce inappropriate finger flexion during treatment. Thus, the position entails problems with degenerative joints that are vulnerable to the stiffness produced by immobilization, which is a common problem in elderly people. In addition, a rise in carpal tunnel pressure from 18 mmHg of neutral position to 47 mmHg of flexion position has been detected, [20] median nerve compression being the most common complication of DRF [21, 22].

### Evaluation of treatment

Treatment outcomes of DRFs can be measured with a variety of tools. Patient-reported outcome measures (PROMs) describe a patient’s view of their symptoms combined with functional status. The tools used in the evaluation measure the mobility and usability of the forearm and wrist and include the Patient Rated Wrist Evaluation (PRWE) questionnaire, the Disabilities of the Arm, Shoulder and Hand (DASH) questionnaire, the Gartland-Werley questionnaire and the Visual Analogue Scale (VAS). A commonly used objective measure is grip strength. In addition, the 15D questionnaire measures the patient’s general quality of life through different questions and takes into account diverse areas of life [23]. The outcomes are indexed and comparable with a reference population and the patient’s own results at different stages of the treatment [24]. In addition to PROM’s, patient characteristics have been shown to be associated with treatment outcome [25]. The severity of acute pain, catastrophic thinking and trauma-related anxiety experienced by the patient are measured using the pain catastrophizing scale (PCS) [26]. The scale has been shown to be associated with finger stiffness after DRF [27], and it is one of the most commonly used tools for measuring the catastrophic thinking related to pain [26].

### Previous studies

The literature on the non-operative treatment of DRFs is extensive. However, previous studies that have examined the most valid method for cast immobilization have been mainly case studies without control groups. The existing comparative trials lack uniform and patient-rated outcome measures, and suffer from inadequate randomizing and short follow-up periods [10–13]. Interestingly, there have been no previous studies that have compared the flexion-ulnar deviation position with the functional cast position in elderly patients. Consequently, the Cochrane review has concluded there is not enough evidence based on randomized controlled trials to conclude which method of cast immobilization is the best for the common types of DRFs in the elderly [28].

It has been shown that the functional outcome of DRF in elderly people (over 64 years) is poorly correlated to the radiographic outcome [29–35]. Moreover, it has been suggested that functional outcome after DRFs in elderly patients may be affected by more than fracture-specific factors. Pain catastrophizing thoughts and a fear of using the injured limb are related to disability, increased pain and muscle weakness in all upper extremity traumas including DRF patients [36–39]. Pain catastrophizing as a predictor of functional outcome has been mostly studied among patients diagnosed with chronic musculoskeletal pain such as osteoarthritis, lower back pain or acute whiplash injuries [40–42]. Pain catastrophizing has not, however, been previously studied in elderly patients with non-operatively treated DRF.

The aim of this pragmatic, randomized controlled study is to compare functional outcome measured with PRWE score after DRF treated with volar flexion-ulnar deviation cast immobilization or functional cast immobilization in elderly patients over 64 years of age. Further, we aim to assess whether PCS and functional outcome after DRFs are associated.

## Methods and design

The present study is a pragmatic, prospective, randomized controlled, multi-centre trial. The trial centres are Tampere University Hospital, Central Finland Central Hospital and Satakunta Central Hospital. The study aims to compare two different cast immobilization positions. The two cast positions compared are slight volar flexion-ulnar deviation immobilization (widely used globally) and functional immobilization (also commonly used in several countries). See Additional files 1, 2, 3 and 4 for the pictures of the cast positions.

The primary outcome in this study is the PRWE score measured after one and 2 years. The secondary outcomes measured are Quick-DASH score, pain in visual analogue scale (VAS), quality of life (15D), grip strength, complications and number of surgical interventions and cast changes. Subgroup analysis will be performed to identify patient-specific features indicating good or poor outcomes and the association of the features with PCS. Fractures will be classified with AO-classification.

### Hypotheses

Our primary hypotheses in the study are as follows:

(i) The volar-flexion, ulnar deviation cast and the functional cast treatments will yield similar functional outcomes measured with PRWE
(ii) The volar-flexion, ulnar deviation cast and the functional cast treatments will result in corresponding outcomes with regard to quality of life and grip strength. Functional cast treatment will result in lower rates of complications and number of cast changes.
(iii) High PCS will be associated with poor functional outcome on the PRWE scale despite the radiographic parameters.
(iv) The high grip strength of the uninjured limb (ie. patient general physiological strength) will not predict the poor functional outcome on the PRWE despite the radiographic parameters.

### Patient selection and methods

The eligible study population comprises conservatively treated elderly patients (over 64 years of age) with a DRF identified in the public or referral emergency departments (ER) of participating hospitals.

The following criteria were used in patient selection:

#### Inclusion criteria

- low energy intra- or extra-articular dorsal primarily stable, reducible DRF within 3 cm of the radiocarpal joint diagnosed with lateral and posterior-anterior radiographs in ER
- physician on-call (general practitioner, acute physician, orthopaedic resident, orthopaedic consultant) thinks patient would be suitable for non-operative treatment

#### Exclusion criteria

- Operative treatment
- Refused to participate in the study
- Open fracture more than Gustilo 1 gradus
- Under 65 years of age
- Chauffeure’s or Barton’s fracture
- Smith’s fracture (volar angulation of the fracture)
- Does not understand written and spoken guidance in local languages
- Pathological fracture or previous fracture in the same wrist, forearm or elbow

### Randomization

All patients will be randomized after diagnosis, but before treatment, to either functional cast or volar flexion-ulnar deviation cast using a random number matrix in block allocation fashion. The blocks will be dependent on age, centre and intra-extra articular fracture because, based on the literature, functional outcome is affected by age and presence of intra-articular fracture [43, 44]. The treatment allocations from the matrix will be sealed in envelopes and situated in the emergency room.

After patient enrolment has been confirmed and informed consent obtained, the patient will be asked to fill in the PCS questionnaire and a medical orderly will open the randomization envelope. The physician on-call will be responsible for the reduction, if needed. The medical orderlies responsible for the casting will not participate in the study in any other way. The research coordinator monitors the study flow and an independent monitoring committee will be established for the study.

### Intervention

If the fracture is well aligned, closed reduction will not be performed. The treating physician will perform closed reduction when necessary, but specific thresholds (shortening, dorsal angulation, radial inclination) is not set. Closed reduction takes place under local anaesthesia by means of a local infiltration commonly used in Finland. The technique of closed reduction is not limited to some specific technique. Fluoroscopy may be used if it is available in the participating centre. Since DRFs are usually treated by healthcare centre physicians, experienced acute physicians and trauma surgeons, we do not see any reason to limit the number, education or experience of the on-call physicians in this pragmatic trial.

The booklet showing cast position examples will be delivered to the participating centres and adequate training will be given to ensure a uniform position of casting in the study. The initial material of the cast is plaster. After reduction, a set of radiographs will be taken to verify the position of the fracture. Should the physician on-call accept that the fracture will be treated non-operatively, the cast allocated will be used for 5 weeks.

### Follow-up

Follow-up visits in this study will be conducted in primary healthcare centres, as seen necessary by the physician on-call. We expect that follow-ups will be arranged according to regional guidelines, normally after 1 and 2 weeks with radiographs. Due to the pragmatic nature of the trial, we will not set any angulation degrees or shortening limits to indicate operative treatment. In case secondary displacement of the fracture occurs during the follow-up, the treating physicians will use their discretion to consult a local orthopaedic surgeon or hand surgeon who will perform the necessary operative treatment. Since patients will be randomized in blocks by the hospital, we assume that randomization will take care of any possible differences between centres.

**Fig. 1:**
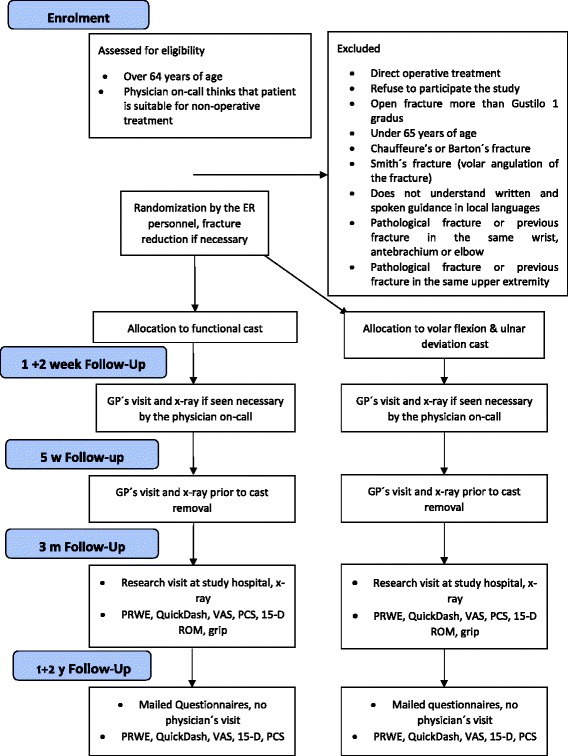
The flow chart of the study

Figure 1 cast immobilization time is estimated to be 5 weeks, according to current guidelines in Finland. The final visit to a general practitioner (GP) will be at the time of cast removal. All patients will undergo an x-ray prior to cast removal. The decision to refer patients to a physiotherapist after cast removal will be left to the discretion of the treating GP.

The first research visit will occur after 3 months at the orthopaedic outpatient clinic in the hospital where the treatment was initially started. This visit is specifically part of the study protocol. During this visit, direct lateral and anteroposterior radiographs will be taken. PRWE, PCS, Quick-DASH, 15D, pain in VAS and grip strength (both hands) will all be assessed. Information on the number of cast changes during treatment will be acquired from the patient.

Primary outcome, PRWE at 1- and 2-year follow-up will be measured by using an Internet-based system. Patients not using the Internet will be contacted by phone or regular mail by a study nurse. Complications and the number of surgical interventions will be recorded on the patient’s medical files after 1 and 2 years (Table 1).

**Table 1:** The assessments and procedures of the trial

	Medical history	Radiograph	PRWE	PCS	Pain	Grip	Quick-DASH	15D	ROM
Baseline	x	x		x					
1–2 weeks	(x)	(x)							
5–6 weeks	(x)	x							
3 months	x	x	x	x	x	x	x	x	x
1 year	x		x	x	x		x	x	
2 years	x		x	x	x		x	x	

### Power analysis

In this trial, a validated wrist specific PRWE score will be used as the main outcome measure. The minimal clinically important difference (MCID) in PRWE is 11 points and standard deviation (SD) is 14 points [45]. In power calculations, we determined the required sample size per group to be 40 patients with 95% confidence interval, power of 0.95 and SD of 14. Thus, in order to have enough statistical power, 40 patients in both groups have to complete 1 year follow-up. Assuming a drop-out rate of 30%, group size would be 57 (114 in total).

### Analysis of the material

All anonymised information gathered in the study will be stored in a study cloud-registry at Tampere University Hospital. The registry is protected with passwords and the data will be deleted 15 years after the end of the study.

### Statistical analysis

Characteristics of the participants will be described using mean and standard deviation, median and quartiles (continuous variables) or proportion (categorical variables). The patients will be analysed according to the intention-to-treat principle, if the patient changes to a different treatment group. Groups at baseline will then be compared using t-test, Mann-Whitney U or Fisher’s exact test. Primary (PRWE) and secondary outcomes (Quick-DASH score, VAS, 15D, grip strength, complications and number of surgical interventions and cast changes) will be compared between the groups at 12 months and 24 months using the Mann-Whitney U test. The results are presented with 95% confidence intervals. Two-way-tables with the chi-square test will be used for dichotomous variables. In subgroup analysis, the effect of age, sex, fracture group, smoking and other diseases will be evaluated against the scores and overall quality of life after fracture. Analysis of covariance will be used to assess the effect of PCS on the outcome of cast treatment. PRWE will be used as a dependent variable, cast as independent and PCS as covariate. The effect of cast treatment on the PRWE is also investigated in multivariable analysis by performing linear regression analysis since the dependent variable, PRWE, is normally distributed. The main explanatory variables are believed to be cast, age, sex, fracture group, smoking and other diseases. Of the authors, AR is responsible for the statistics.

### Ethics

The trial protocol and additional papers, including consent form, patient information sheet and questionnaires have been approved by the Regional Ethics Committee of Tampere University Hospital (Approval number: R16035).

### Time schedule

The recruitment for the study began in August of 2016 and the results will be analysed after the 1 and 2-year follow-up period. The final report will be published by the end of 2019.

## Discussion

This publication presents a prospective, randomized, national multi-centre trial of the non-operative treatment of DRF. It depicts details of the patient flow, randomization, intervention, follow-up and analysis of the material.

The limitations of this study are the lack of patient’s blinding to treatment and the exclusion of patients with other than dorsally displaced DRFs. The blinding for treatment is not feasible for practical reasons and by excluding all patients other than dorsally displaced DRF patients from the study enables us to compare our results to other trials.

The strengths of the study are the pragmatic nature of the study, the comprehensiveness of Finnish registers with personal identification number and the excellent coverage of our study hospitals. The pragmatic nature of the study, including no pre-set thresholds for surgical treatment in case of secondary displacement, reveals the real impact of the treatment of DRFs in the elderly population. The registers allow us to follow-up patients during the study, and therefore reduces the probability of patients being lost in follow-up. All surgical interventions, for example, are recorded to registers regardless of the hospital. Other strengths of the study are the validated primary outcome measure (PRWE) and the taking into account of pain catastrophizing tendency in explaining the results of the treatment.

## Additional files

Additional file 1: Figure S1. The first picture of flexion-ulnar deviation cast. (TIFF 35156 kb)

Additional file 2: Figure S2. The second picture of flexion-ulnar deviation cast. (TIFF 35156 kb)

Additional file 3: Figure S3. The first picture of functional cast. (TIFF 35156 kb)

Additional file 4: Figure S4. The second picture of functional cast. (TIFF 35156 kb)

## Abbreviations

15-D: 15 dimensional health-related quality of life indicator
DRF: distal radius fracture
GP: general practitioner
PA: posterior-anterior
PCS: Pain Catastrophizing Scale
PRWE: Patient Rated Wrist Evaluation
Quick-Dash: Quick Disability of Arm, Shoulder and Hand
VAS: visual analog scale
