# Supplementary figures and images for: Comparison of volar-flexion, ulnar-deviation and functional position cast immobilization in the non-operative treatment of distal radius fracture in elderly patients: a pragmatic randomized controlled trial study protocol
Source: BMC Musculoskelet Disord. 2017 Sep 18;18:401. doi: 10.1186/s12891-017-1759-y (PMC5604291; doi:10.1186/s12891-017-1759-y)

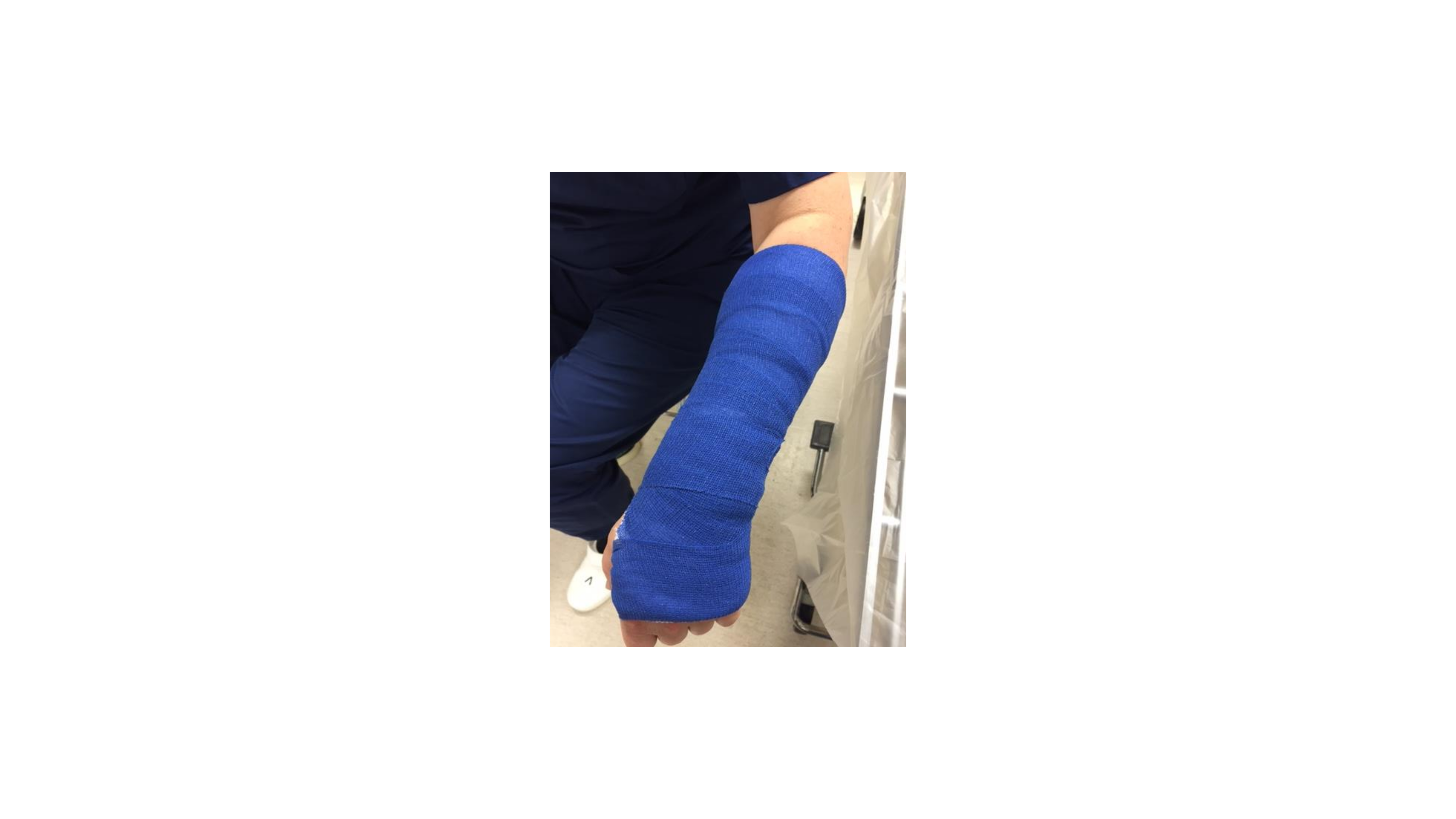

Supplement: Supplementary file 1 — The first picture of flexion-ulnar deviation cast. (TIFF 35156 kb) [file 12891_2017_1759_MOESM1_ESM.tif]

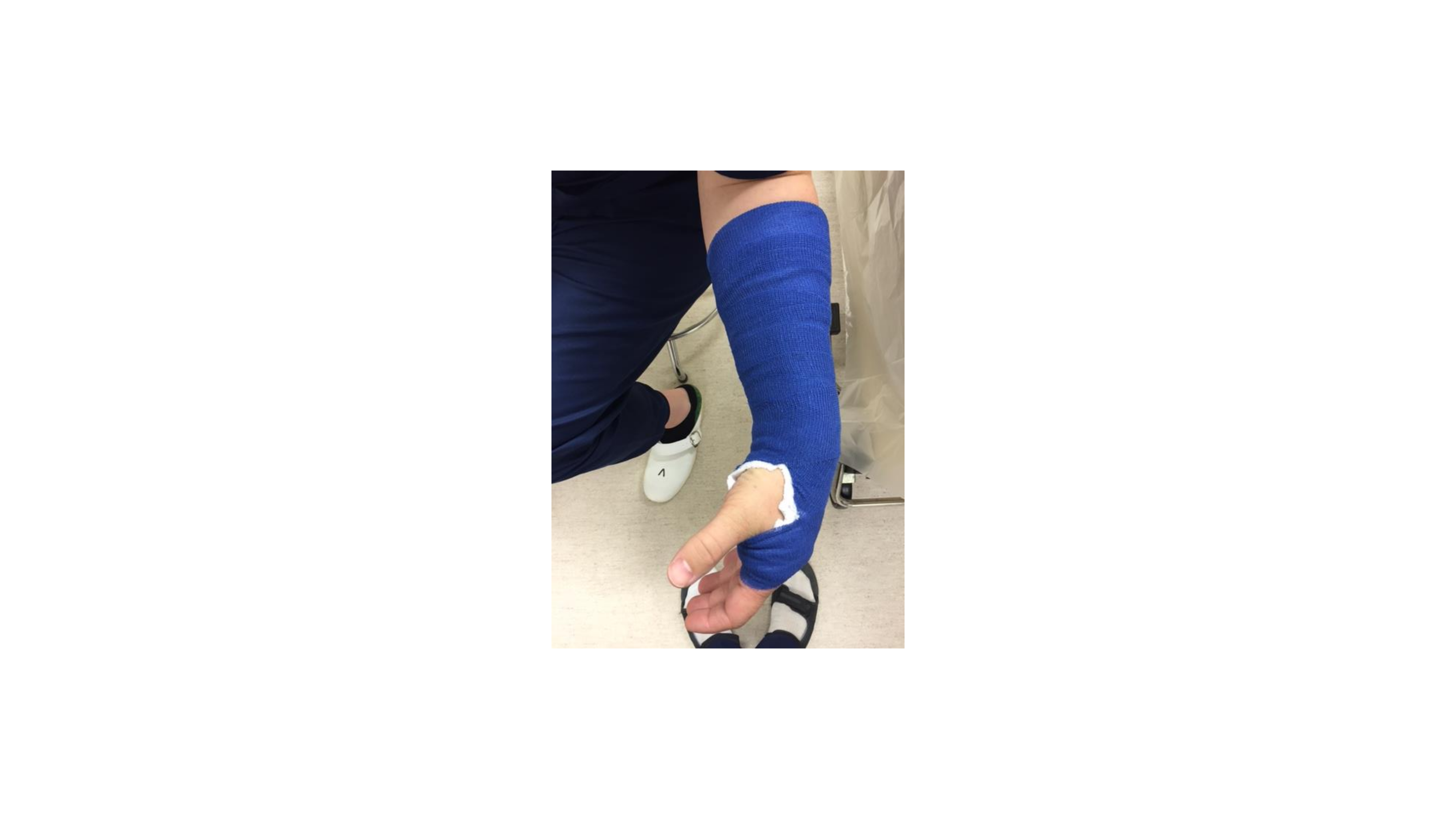

Supplement: Supplementary file 2 — The second picture of flexion-ulnar deviation cast. (TIFF 35156 kb) [file 12891_2017_1759_MOESM2_ESM.tif]

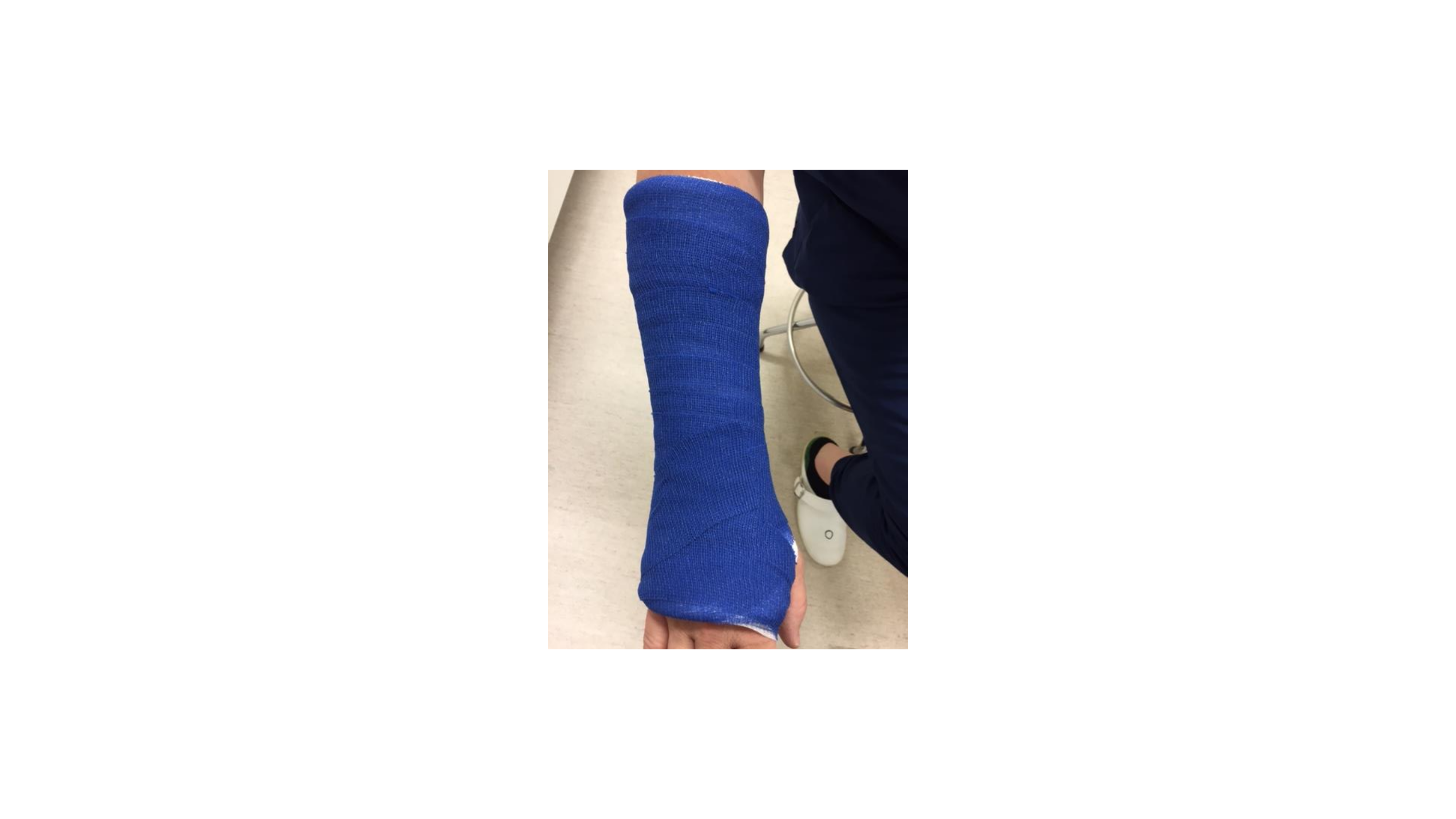

Supplement: Supplementary file 3 — The first picture of functional cast. (TIFF 35156 kb) [file 12891_2017_1759_MOESM3_ESM.tif]

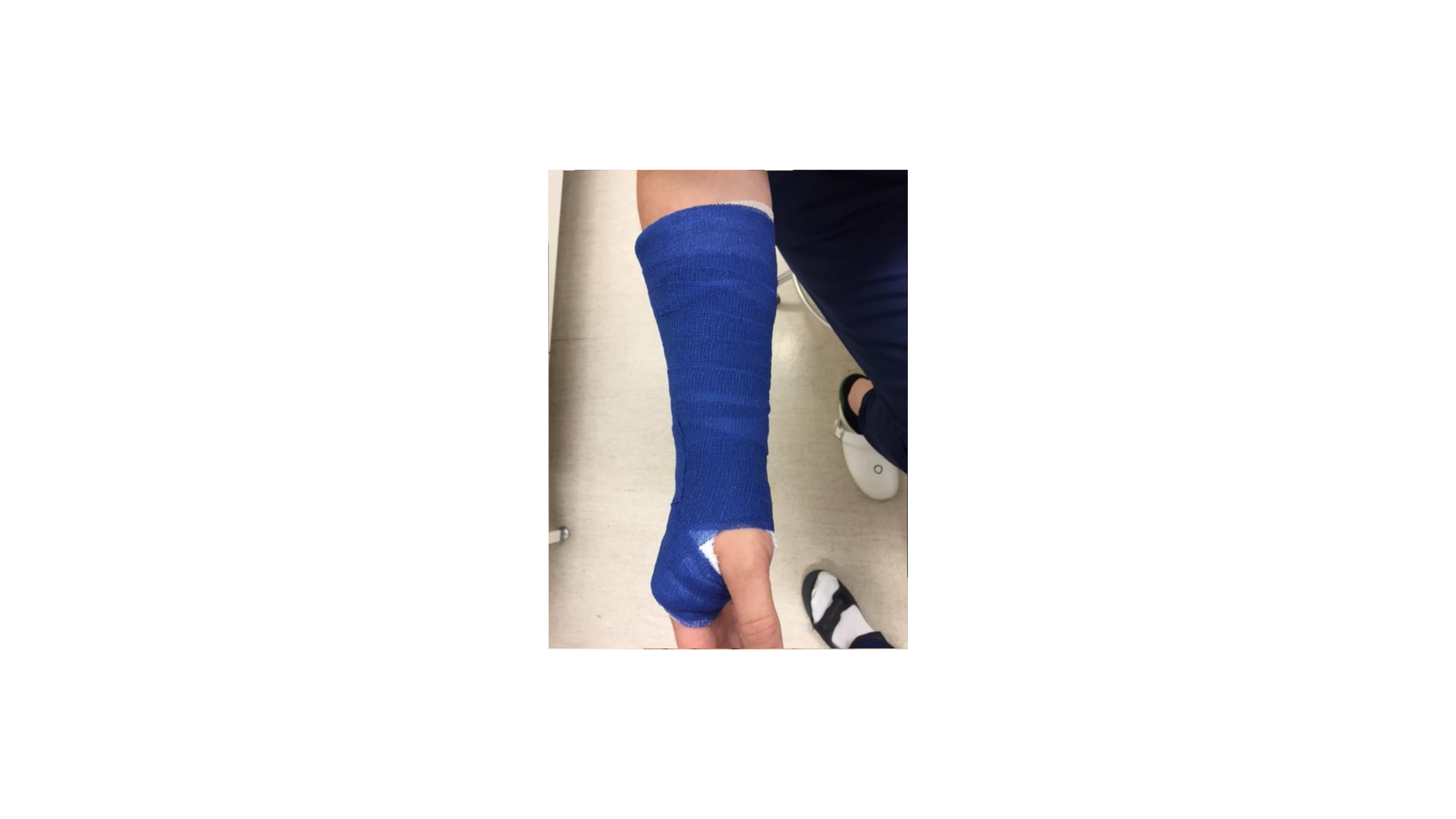

Supplement: Supplementary file 4 — The second picture of functional cast. (TIFF 35156 kb) [file 12891_2017_1759_MOESM4_ESM.tif]
